# Assessing Medical Students' Perspectives on Organ Donation: A Cross-Sectional Study

**DOI:** 10.7759/cureus.63556

**Published:** 2024-07-01

**Authors:** G Vinay, Mangala Gowri SR, Muralidhar Reddy Sangam, Praveen K, Raju R Bokan, Roonmoni Deka, Amandeep Kaur

**Affiliations:** 1 Anatomy, All India Institute of Medical Sciences, Guwahati, Guwahati, IND; 2 Physiology, Prathima Relief Institute of Medical Sciences, Warangal, IND; 3 Anatomy, All India Institute of Medical Sciences, Rishikesh, Rishikesh, IND

**Keywords:** awareness, attitude, medical students, knowledge, organ donation

## Abstract

Background

Organ donation is a community service that not only saves lives but also improves the quality of life. The major concerns causing organ shortage in the country are the lack of awareness and correct knowledge among the public and myths and misconceptions clouding organ donation because of religious and cultural barriers.

Material and methods

A cross-sectional study was conducted among 300 medical students of a private medical college in the Telangana region, India, from July 2019 to October 2019 after approval from the Institutional Ethics Committee (IEC). A total of 300 participants (218 females (72.66%) and 82 males (27.33%)) were included in the study. Respondents completed a three-section questionnaire that included sociodemographic data, 15 questions on knowledge, and 12 questions on the attitude and ethical aspects of organ donation. Statistical tests utilized for investigation were the Student's t-test and one-way ANOVA to compare knowledge and attitude scores.

Results

The mean knowledge score among the participants was 10.85±1.79, with a P value of 0.45. The mean attitude score concerning organ donation among the participants was 45.5±4.47, with a P value of 0.44. The majority (87.1%) showed a positive attitude in this study.

Conclusions

The study emphasizes the necessity of interdisciplinary educational interventions for medical students to help them realize the complexities of the problem holistically. Their attitude regarding organ donation is not significantly affected by traditional educational interventions such as lectures and demonstrations. Educated healthcare professionals will play a critical role in motivating the public for the cause of organ donation promotion.

## Introduction

Organ donation is a noble deed as it saves many lives and improves the quality of life of many more. However, there is a major shortfall in the availability of organs. This leads to potentially preventable death and morbidity in many people [1]. Both living and deceased persons can donate organs. Damaged and nonworking organs because of injury or chronic diseases can be supplanted by giving organs, which will increase their life span and quality of life [2].

Organ donation is yet to gain momentum in India. The organ donation program will be successful if the general public is made aware and educated about it [3]. Lawfully, the donation of organs is authorized by the Government of India through the Transplantation of Human Organs Act of 1994 [4]. The huge difference in organ demand and supply is a global issue and can be credited to multiple reasons. The significant worries causing organ deficiency in India are because of lack of awareness and knowledge among the community, mythology, and delusion clouding organ contribution because of spiritual and social barriers [5].

Medical practitioners have an important role in conveying the right information and removing obstacles regarding organ donation among the general population as they are the primary persons to make an association with a potential donor's family [6]. Medical students, who will be future general practitioners, are the first contact with the patients and their families and help create awareness regarding organ donation. Thus, enhancing their knowledge and attitude through changes in the medical curriculum, and providing educational programs and role plays are crucial [7].

A positive attitude promotes a growth mindset, encouraging students to embrace challenges and learn from failures. This mindset enhances their ability to acquire new knowledge and adapt to different subjects [8]. Medical professionals' knowledge, attitudes, and practices are vital in promoting an atmosphere that positively impacts organ donation and procurement rates [9].

The need of the hour is to enhance awareness among the general population, starting with healthcare students who will be future practitioners, and only a few studies related to awareness, ethical issues, and attitudes of medical undergraduates have been conducted. The present study was done to assess the knowledge and attitude about organ donation among the medical students of the Khammam region, Telangana, India. This may help future doctors impart the proper knowledge and eliminate the barriers regarding organ donation among the public.

Objectives

First, this study aimed to assess and compare knowledge and attitudes concerning organ donation among medical students. Second, it aimed to compare the knowledge scores and attitude scores among male and female students.

## Materials and methods

The research was conducted among medical students studying in the second, third, and fourth years of their MBBS (Bachelor of Medicine and Bachelor of Surgery) at Mamata Medical College, Khammam, Telangana, India. Sample size calculation was done using the formula 4pq/d^2, where p was 0.125 (prevalence regarding organ donation from a previous study done by Ramandeep et al. 2016 [10]), q was 0.875, and d was 0.05. The sample size was computed as 175 and increased to 300 for efficient results.

Out of the 300 participants, 218 (72.66%) were females and 82 (27.33%) were males. Participants' mean age was 20.32 years, and the range was 18-24 years. The participants were divided into three groups based on the year of Bachelor of Medicine and Bachelor of Surgery (MBBS). The second-year students were 111 (37%), the third year were 98 (32.66%), and the fourth year were 91 (30.33%).

The study was a cross-sectional study conducted among 300 medical students who gave informed consent. The anonymity and confidentiality of respondents were maintained, and participation was voluntary. This study was conducted from July 2019 to October 2019. The participants of the age group from 18 to 25, belonging to the second, third, and fourth years of MBBS were included in this study. The participants in the first year of the MBBS and those who remained absent from the study were excluded.

After receiving approval from the Institutional Ethics Committee (IEC/IRB No.26/MMC/2019), data were collected using a pre-validated questionnaire created using Google Forms and distributed via email. The questionnaire was organized into three sections. The first section focused on understanding the respondent’s general information, including their sociodemographic profile.

The second section was designed to assess the knowledge of the respondents about organ donation. There were fifteen pre-validated questions in the questionnaire to assess the knowledge of the respondents regarding organ donation. Three options, “True,” “False,” and “don’t know,” were provided under each question for the respondents to answer. A self-constructed cumulative knowledge score was prepared by the authors by adding the number of correct answers (1 point per correct reply and 0 points for incorrect or “don’t know”). The maximum score for knowledge was 15. Scores of 12 to 15 were considered as good knowledge, 8 to 11 as average knowledge, and less than 7 as poor knowledge. The mean of the sample came to be 10.85 with a standard deviation of 1.79.

The third section consists of six clinical scenarios, each carrying two sub-questions for attitude and ethical aspects of organ donation, which were based on a five-point Likert scale with scores ranging from 5 (strongly agree) to 1 (strongly disagree). A score of 35 is considered neutral; higher than 35 indicates a good attitude, and lower than 35 indicates a negative attitude.

Statistical analysis

Data obtained were entered into an MS Excel spreadsheet (Microsoft® Corp., Redmond, WA), and data analysis was done with the help of Statistical Product and Service Solutions (SPSS) (IBM SPSS Statistics for Windows, Armonk, NY). Descriptive statistics were used to describe data frequency and percentages. Student’s t-test and one-way ANOVA were used to compare knowledge and attitude scores among different groups. P < 0.05 was considered statistically significant.

## Results

The questionnaires were distributed to 300 medical students, of which 111 participants were from the second year, 98 from the third year, and 91 from the fourth year of MBBS. Results were considered significant if the associated P value was less than 0.05. Participants' mean age was 20.32 years (range 18-24 years), 218 were females (72.66%), and 82 were males (27.33%).

The mean knowledge score among the participants was 10.85±1.79, with a P value of 0.45. Fourteen students (4.6%) had poor knowledge (less than or equal to 7 out of 15), and 118 (39.4%) participants had good knowledge (more than or equal to 12 out of 15) regarding organ donation.

**Figure 1:**
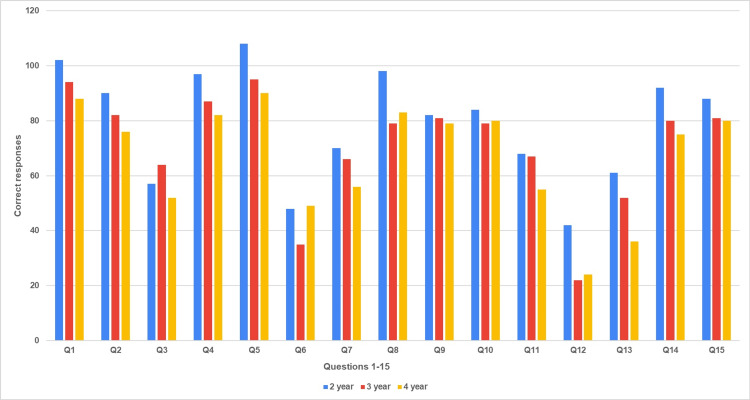
Correct responses to Questions 1–15 year-wise distribution.

The correct responses received from the participants for Questions 1-15 are shown in Figure 1. 

The mean score of attitude concerning organ donation among the participants was 45.5 ± 4.47, with a P value of 0.44. A score of 35 is considered neutral; more than 35 is a positive attitude, and less than 35 is a negative attitude. Only 9.3% of participants had a negative attitude toward organ donation. Approximately 3.6% were neutral, and 87.1% of participants showed a positive attitude toward organ donation.

The following table shows the mean age of the participants as well as the mean scores of knowledge and attitude about organ donation of second-, third-, and fourth-year MBBS students. As shown in Table 1, the knowledge score was highest in fourth-year students and least in second-year students. The attitude scores were higher in second-year students and least in third-year students. 

**Table 1:** Mean age and mean scores of knowledge and attitude concerning organ donation of second-, third-, and fourth-year students of Bachelor of Medicine and Bachelor of Surgery (MBBS).

Year of MBBS	Age (years)	Knowledge scores	Attitude scores
2^nd^ Year (111)	19.35 ± 0.85	10.75 ± 2.08	46.01 ± 4.43
3^rd^ Year (98)	20.37 ± 0.92	10.78 ± 1.62	44.65 ± 4.46
4^th^ Year (91)	21.44 ± 0.88	11.04 ± 1.58	45.79 ± 4.45
Total (300)	20.32 ± 1.23	10.85 ± 1.79	45.50 ± 4.47

Table 2 shows the comparison of the mean values of knowledge and attitude scores among the male and female participants. As observed in Table 2, the knowledge scores of the female participants were higher when compared to male participants, but the attitude scores were higher in males in comparison to females. However, the knowledge and attitude scores difference between males and females were statistically not significant. There was a weak positive association between knowledge and attitude scores in the study group.

**Table 2:** Mean knowledge and attitude scores in males and females.

Variables	Males (82)	Females (218)	t-value	P value
Knowledge scores	10.72 ± 1.92	10.90 ± 1.74	0.27	0.45
Attitude scores	45.82 ± 5.26	45.38 ± 4.14	0.77	0.44

## Discussion

This cross-sectional study conducted among 300 undergraduate medical students of Mamata Medical College, Khammam, Telangana, India, assessed their knowledge and attitudes toward organ donation. The mean age of the study population was 20.32±1.23 years, similar to surveys done by Alex et al. [11] and Shireen et al. [12], who accepted medical undergraduates as participants. The mean knowledge score was 10.85±1.79, and the members with good knowledge scores were 39.4%. Comparative discoveries were noted by Sindhu et al. [13] and 56% in the investigation of Kishore et al. [14].

Organ donation is defined as “When a person allows an organ of theirs to be removed, legally, either by consent while the donor is alive or after death with the assent of the next of kin.” Common transplantations after organ donations include kidney, heart, liver, pancreas, intestines, lungs, bones, bone marrow, skin, and cornea [15]. Despite the country’s huge population, the organ donation rate in India is far lower than in Western nations. This is because of certain sociocultural beliefs and traditions that restrain the donation of organs from the living individual as well as from the corpse.

In our study, 57.5% of the participants were aware of the act regulating the procedure of organ donation. This is higher compared to the study conducted by Ali et al. [16] in Karachi, where only 13.3% of the medical undergraduates were aware of the act, which is similar to the study conducted by Vinay et al. [17] in Mangalore, where 13.9% of medical undergraduates were aware of the act. Forty-four percent of undergraduates did not believe that chronic ailments such as diabetes mellitus and cardiovascular illnesses are contraindications for organ donation, which is higher when compared with 26.5% of Marques et al. [18] and 18% as reported by Srinivas et al. [19].

Organ donation will not deform the body or meddle with funeral arrangements. The current study showed 85% of the participants realized it would not cause distortion, which was in line with the study done by Kishore et al. [14], where 88% of the participants agreed, though it was just 57% as reported by Srinivas et al. [19]. There is no set age limit for organ donation. Marques et al. [18] found that 23% of the participants felt that the information regarding this topic was inadequate. Every religion would support and consider organ donation a blessing. In the present study, 83% of the study group agreed with it. This was similar to research conducted by McGlade et al. [20], where 82% of respondents agreed, 62% according to Sahana et al. [21], and 81% by Srinivas et al. [19].

In our study, 87% of the students showed a positive attitude, similar to the study by Vijayan et al. [22], where 89% showed a positive attitude, and in the study by Vinay et al. [17], 91% showed a positive attitude. This is higher compared to the Polish study by Mikla et al. [23], where only 65.44% showed a positive attitude. Males exhibited a more positive attitude than females, which is in line with studies done by Vinay et al. [17] and Adithyan et al. [3].

Limitations

The study population represents only a small fraction of the medical community, so the results cannot be generalized to the whole community. Further studies including a larger sample size need to be conducted to validate the results.

## Conclusions

Our study concludes that a large portion of the medical undergraduates had adequate knowledge and a positive attitude toward organ donation. The knowledge about the act of managing organ donation, which is considered basic for specialists, was found to be poor. The present study found no statistically significant association between knowledge and attitudes toward organ donation. Medical students have a distinct role in organ donation, being the future torchbearers. Currently, there is no conventional teaching aimed at organ donation, including its moral aspects. If motivated, they can create awareness, inspire the general population, and promote organ donation in the community, which is the need of the day.

## Appendices

Appendices

**A.** **Sociodemographic data**

Age (years):

Gender:

Year of MBBS:

B. Semi-structured questionnaire

**Table 3:** Knowledge of medical students towards organ donation

Sl no	Questionnaire	True	False	Don’t know
1	Organs can be donated after death			
2	Having an infectious disease is a contraindication to being an organ donor			
3	There is no act/law for organ donation			
4	There is a short­age of organs in India			
5	Organ transplants are successful in prolonging and improving the quality of a recipient's life			
6	Having a cardiovascular condition or diabetes mellitus is a contraindication to becoming an organ donor			
7	Is it an offense to accept money or any other benefits for organ donation			
8	Patients on the waiting list who are in critical condition have priority over other patients			
9	Organ donation after death causes disfigurement to the donor			
10	Persons who have experienced irreversible brain death but maintained on a life-support system can be considered potential organ donors.			
11	Medical insurance can be extended to include organ transplant surgery.			
12	The age of organ donors can range from 28 days to 70 years.			
13	If a deceased patient has signed an organ donor card but the family does not wish to donate the organs, the hospital is required to honor the wishes of the family.			
14	When an organ is donated, the family of the donor pays for the surgery.			
15	The approach for organ donation may be made to every religious group.			

C. Semi-structured (clinical scenarios) questionnaire related to attitude, consent, and ethical aspects of organ donation

Please rate the extent you agree or disagree with each statement

Case 1) Mr X, a lawyer by profession has decided to donate his organs to the needy after his death. Rate the following questions:

1) Each and every individual like Mr. X has the right to decide their willingness toward donating organs

a) Strongly agree   b) agree   c) neutral   d) disagree   e) strongly disagree

2) If the family members of Mr. X do not wish to donate his organs then organ donation can’t be done

a) Strongly agree   b) agree   c) neutral   d) disagree   e) strongly disagree

Case 2) Mr Y 19-year-old male met with a road traffic accident caused a head injury and was brought by police personnel in an ambulance to RP hospital. He was on a ventilator and later declared brain-dead by an ICU consultant.

3) If an approach is made to family members of brain-dead patient for organ donation, they will be upset

a) Strongly agree   b) agree   c) neutral   d) disagree   e) strongly disagree

4) In this case it is mandatory consent must be obtained by family members of patients

a) Strongly agree   b) agree   c) neutral   d) disagree   e) strongly disagree

Case 3) Mr A is suffering from renal failure and Mr B is suffering from unilateral corneal blindness, priority of organ donation is given to Mr A

5) The decision taken in the above scenario is correct

a) Strongly agree   b) agree   c) neutral   d) disagree   e) strongly disagree

6) The identity of the donor can be revealed to the recipient family

a) Strongly agree   b) agree   c) neutral   d) disagree   e) strongly disagree

Case 4) 25-year-old Mr X is willing to donate one of his kidneys to the patient with chronic renal failure who is very much need for organ transplantation.

7) The decision taken by Mr X must be respected

a) Strongly agree   b) agree   c) neutral   d) disagree   e) strongly disagree

8) The intention behind organ donation is to save the lives of others

a) Strongly agree   b) agree   c) neutral   d) disagree   e) strongly disagree

Case 5) Mr Y an HIV patient wants to donate his organs

9) HIV patients can donate the organs

a) Strongly agree   b) agree   c) neutral   d) disagree   e) strongly disagree

10) Recipient should be made aware of the information that donor is HIV positive

a) Strongly agree   b) agree   c) neutral   d) disagree   e) strongly disagree

Case 6) A 60-year-old male patient is suffering from cirrhosis of liver, the only treatment left is liver transplant. So his son aged 25 years decided to donate liver to his father

11) It is the duty of the son to save his father by donating his organ

a) Strongly agree   b) agree   c) neutral   d) disagree   e) strongly disagree

12) This decision taken by the son due to emotional bonding with his father should be considered

a) Strongly agree   b) agree   c) neutral   d) disagree   e) strongly disagree

## References

[1] Wilkinson D, Savulescu J (2012). Should we allow organ donation euthanasia? Alternatives for maximizing the number and quality of organs for transplantation. Bioethics.

[2] Sadala ML, Mendes HW (2000). Caring for organ donors: the intensive care unit nurses' view. Qual Health Res.

[3] Adithyan GS, Mariappan M, Nayana KB (2017). A study on knowledge and attitude about organ donation among medical students in Kerala. Indian J Transplant.

[4] (2024). The transplantation of human organs and tissues act no. 42 of 1994. https://www.indiacode.nic.in/bitstream/123456789/1962/1/199442.pdf.

[5] Panwar R, Pal S, Dash NR, Sahni P, Vij A, Misra MC (2016). Why are we poor organ donors: a survey focusing on attitudes of the lay public from northern India. J Clin Exp Hepatol.

[6] Schaeffner ES, Windisch W, Freidel K, Breitenfeldt K, Winkelmayer WC (2004). Knowledge and attitude regarding organ donation among medical students and physicians. Transplantation.

[7] Mekahli D, Liutkus A, Fargue S, Ranchin B, Cochat P (2009). Survey of first-year medical students to assess their knowledge and attitudes toward organ transplantation and donation. Transplant Proc.

[8] Ogut E, Senol Y, Yildirim FB (2017). Do learning styles affect study duration and academic success?. Eur J Anat.

[9] Bardell T, Childs AL, Hunter DJ (2002). Organ donation: a pilot study of knowledge among medical and other university students. Ann R Coll Physicians Surg Can.

[10] Kaur Ramandeep, Begum Nilavansa S, Kaur Amritpal (2015). A quasi experimental study to assess the effectiveness of structured teaching programme on knowledge and attitude regarding organ donation among young adults in selected colleges of Jalandhar, Punjab 2014. Asian J Nur Edu Res.

[11] Alex P, Kiran KG, Baisil S, Badiger S (2017). Knowledge and attitude regarding organ donation and transplantation among medical students of a medical college in South India. Int J Community Med Pub Health.

[12] Shireen N, Ansari MW, Indupalli AS, Selladurai S, Reddy SS (2018). Knowledge and attitude about organ donation and transplantation among students of a medical college in Kalaburagi. Nat Jr of Comm Med.

[13] Sindhu A, Ramakrishnan TS, Khera A, Singh G (2017). A study to assess the knowledge of medical students regarding organ donation in a selected college of Western Maharashtra. Med J DY Patil Univ.

[14] Kishore Y, Jothula Jothula, Sreeharshika D (2018). Study to assess knowledge, attitude and practice regarding organ donation among interns of a medical college in Telangana, India. Int J Community Med Public Health.

[15] Tamuli RP, Sarmah S, Saikia B (2019). Organ donation - "attitude and awareness among undergraduates and postgraduates of North-East India". J Family Med Prim Care.

[16] Ali NF, Qureshi A, Jilani BN, Zehra N (2013). Knowledge and ethical perception regarding organ donation among medical students. BMC Med Ethics.

[17] Vinay KV, Beena N, Sachin KS, Praveen S (2016). Changes in knowledge and attitude among medical students towards organ donation and transplantation. Int J Anat Res.

[18] Marqués-Lespier JM, Ortiz-Vega NM, Sánchez MC, Sánchez MC, Soto-Avilés OE, Torres EA (2013). Knowledge of and attitudes toward organ donation: a survey of medical students in Puerto Rico. P R Health Sci J.

[19] Ganta SR, Pamarthi K, K. K. LP (2018). Knowledge and attitude regarding organ donation and transplantation among undergraduate medical students in North coastal Andhra Pradesh. Int J Community Med Public Health.

[20] McGlade D, Pierscionek B (2013). Can education alter attitudes, behaviour and knowledge about organ donation? A pretest-post-test study. BMJ Open.

[21] Sahana BN, Sangeeta M (2015). Knowledge, attitude and practices of medical students regarding organ donation. Int J Cur Res Rev.

[22] Vijayan L, Deepa P (2018). Structured enlightenment program on knowledge and attitude regarding organ donation. MJNHS.

[23] Mikla M, Rios A, Lopez-Navas A (2015). Factors affecting attitude toward organ donation among nursing students in Warsaw, Poland. Transplant Proc.

